# Predation risk and abiotic habitat parameters affect personality traits in extremophile populations of a neotropical fish (*Poecilia vivipara*)

**DOI:** 10.1002/ece3.3165

**Published:** 2017-07-18

**Authors:** Carolin Sommer‐Trembo, Ana Cristina Petry, Guilherme Gomes Silva, Sebastijan Martin Vurusic, Jakob Gismann, Jasmin Baier, Sarah Krause, Julia de Araujo Cardoso Iorio, Rüdiger Riesch, Martin Plath

**Affiliations:** ^1^ College of Animal Science and Technology Northwest A&F University Yangling China; ^2^ Department of Ecology and Evolution Goethe University Frankfurt Frankfurt am Main Germany; ^3^ Núcleo em Ecologia e Desenvolvimento Sócioambiental de Macaé Universidade Federal do Rio de Janeiro Macaé Brazil; ^4^ BSc Study Program “Saude Ambiental” Universidade Federal de Uberlândia Uberlândia Brazil; ^5^ School of Biological Sciences Royal Holloway, University of London Egham UK

**Keywords:** animal personality, behavioral syndromes, boldness, shoaling, water transparency

## Abstract

Understanding whether and how ambient ecological conditions affect the distribution of personality types within and among populations lies at the heart of research on animal personality. Several studies have focussed on only one agent of divergent selection (or driver of plastic changes in behavior), considering either predation risk or a single abiotic ecological factor. Here, we investigated how an array of abiotic and biotic environmental factors simultaneously shape population differences in boldness, activity in an open‐field test, and sociability/shoaling in the livebearing fish *Poecilia vivipara* from six ecologically different lagoons in southeastern Brazil. We evaluated the relative contributions of variation in predation risk, water transparency/visibility, salinity (ranging from oligo‐ to hypersaline), and dissolved oxygen. We also investigated the role played by environmental factors for the emergence, strength, and direction of behavioral correlations. Water transparency explained most of the behavioral variation, whereby fish from lagoons with low water transparency were significantly shyer, less active, and shoaled less than fish living under clear water conditions. When we tested additional wild‐caught fish from the same lagoons after acclimating them to homogeneous laboratory conditions, population differences were largely absent, pointing toward behavioral plasticity as a mechanism underlying the observed behavioral differences. Furthermore, we found correlations between personality traits (behavioral syndromes) to vary substantially in strength and direction among populations, with no obvious associations with ecological factors (including predation risk). Altogether, our results suggest that various habitat parameters simultaneously shape the distribution of personality types, with abiotic factors playing a vital (as yet underestimated) role. Furthermore, while predation is often thought to lead to the emergence of behavioral syndromes, our data do not support this assumption.

## 1 INTRODUCTION

Individual variation in behavioral tendencies that is consistent over time and across contexts—also referred to as animal personality (AP)—has been reported for a multitude of species, including both vertebrates and invertebrates (reviewed in Gosling & John, 1999; Gosling, 2001; Réale, Reader, Sol, McDougall, & Dingemanse, 2007; Bell, Hankison, & Laskowski, 2009). AP is a major component of intraspecific phenotypic variation that integrates genomic and environmentally induced variation (Van Oers et al. 2005; Dingemanse, Kazem, Réale, & Wright, 2010; Freund et al., 2013). Five personality traits received most attention in the literature on AP, namely boldness, exploration, aggression, activity, and sociability (Réale et al., 2007), and ambient predation pressure is thought to be one of the key environmental triggers and selective agents shaping differences among populations in those traits (e.g., Álvarez & Bell, 2007; Archard & Braithwaite, 2011; Brown, Jones, & Braithwaite, 2005; Magurran & Seghers, 1991, 1994; Magurran, Seghers, Carvalho, & Shaw, 1992). For example, Brown et al. (2005) compared populations of the poeciliid fish *Brachyrhaphis episcopi* from four rivers in Panama that either experienced high predation (downstream of waterfalls) or low predation in upstream portions of the streams. In all four rivers, individuals from high‐predation sites were bolder than those from low‐predation stream portions. Likewise, guppies (*Poecilia reticulata*) from high‐predation sites on Trinidad were more willing to feed under predation hazard (Fraser & Gilliam, 1987) and emerged sooner from shelter—a common approach to quantify boldness (e.g., Brown et al., 2005; Polverino, Ruberto, Staaks, & Mehner, 2016; Wilson & Godin, 2009)—than individuals from low‐predation sites (Harris, Ramnarine, Smith, & Pettersson, 2010). Several studies also reported population differences in other personality traits like shoaling/sociability (e.g., *P. reticulata*: Seghers, 1973, 1974; *Phoxinus phoxinus*: Magurran, 1986), activity (*B. episcopi*: Archard & Braithwaite, 2011), and exploration tendencies (*B. episcopi*: Archard & Braithwaite, 2011) among fish populations exposed to varying degrees of predation risk.

Even though several studies demonstrated that not only predation pressure but also various other environmental factors influence personality traits in fish (e.g., habitat structure: Kobler, Maes, Humblet, Volckaert, & Eens, 2011; temperature: Biro, Beckmann, & Stamps, 2010; light intensity/turbidity: Kelley, Phillips, Cummins, & Shand, 2012; Borner et al., 2015), surprisingly few studies have made an attempt to disentangle the relative contributions of different biotic and abiotic ecological factors for the emergence of population differences in personality traits. Indeed, most studies investigating the influence of environmental factors on population differences in AP in fish focused on only one environmental factor (e.g., Archard & Braithwaite, 2011; Brown et al., 2005; Fraser & Gilliam, 1987; Harris et al., 2010), while Brydges, Colegrave, Heathcote, and Braithwaite (2008) found that the interaction between predation risk and habitat stability but not predation alone predicted differences in boldness among populations of three‐spined stickleback (*Gasterosteus aculeatus*). This approach is clearly prone to overlook complex patterns of environmentally induced population differences in AP, where a multitude of ecological factors simultaneously drive divergence in population means of different personality traits. Here, we exemplify how an array of abiotic and biotic habitat parameters affects AP in the neotropical freshwater fish *Poecilia vivipara*. Specifically, we compared six populations inhabiting different coastal lagoons that vary substantially not only in predation risk but also in salinity (from oligo‐ to hypersaline: Caliman et al., 2010), as well as in water transparency, and dissolved oxygen (Table 1). We measured boldness (assessed as time to emerge from shelter and enter an unknown area; Brown et al., 2005; Harris et al., 2010; Polverino et al., 2016; Wilson & Godin, 2009), activity in an open‐field test (Archard & Braithwaite, 2011; Biro et al., 2010; Burns, 2008; Moretz, Martins, & Robison, 2007), and shoaling/sociability (assessed as the time spent in the vicinity of a shoal; Plath & Schlupp, 2008; Ward, Thomas, Hart, & Krause, 2004; Wright & Krause, 2006) of adult female *P. vivipara* from the different lagoons. Our first question was whether populations differ in mean boldness, activity, and shoaling tendencies and whether these differences can be related to the observed variation in the aforementioned environmental factors.

**Table 1:** Differences in abiotic ecological factors and predation risk of the six coastal lagoons in and around the Restinga de Jurubatiba National Park in which female peacock mollies (*Poecilia vivipara*) were collected

Lagoon	Salinity (ppt)	Water transparency^a^^,^ b	DO (mg/L)	Predation level^a^
Catingosa	36.4	Low	8.5	Low
Garças	20.5	High	4.6	Low
Preta	14.0	High	8.4	Low
Carapebus	13.4	High	9.7	High
Imboassica	0.40	Low	9.7	High
Cabiunas	0.20	High	6.9	High

a After Di Dario et al. (2013).

b After Caliman et al. (2010).

Our second question was to what extent populations change mean values of the three personality traits under altered environmental conditions. Ambient environmental conditions can change abruptly within an individual's lifetime, and variable adjustment of personality‐related behavioral traits could be favored by selection (Dingemanse et al., 2010), especially in ecologically flexible species like *P. vivipara*. We simulated altered ecological conditions by collecting females from four of the six lagoons and maintaining them under uniform laboratory conditions—that is, without predator exposure, and under “benign” abiotic conditions—for at least 3 months before testing them as described above.

We used the same datasets from the wild‐caught and laboratory‐maintained cohorts of test subjects to answer our third question, which was related to the occurrence of “behavioral syndromes.” The term was originally used as a synonym for AP (Bell, 2007; Sih, Bell, & Johnson, 2004) and was used to describe correlations of the same behavioral trait across different situations (e.g., correlations of aggressiveness toward conspecifics and toward a predator; Pruitt, Riechert, & Jones, 2008), but usage of this term has more recently changed to describe correlations between different behavioral traits (e.g., correlations between boldness and activity or boldness and sociability, e.g., Mazué, Dechaume‐Moncharmont, & Godin, 2015). We addressed the role of biotic and abiotic habitat parameters for shaping the strength and direction of syndrome structures. There is evidence that behavioral syndrome structures (both within and across populations) can become stronger as predation pressure increases (Bell, 2005; Bell & Sih, 2007; Dingemanse et al., 2007), one possible explanation being that selection from predation favors distinct correlations of behaviors, for example, if active individuals with high shoaling tendencies have a higher likelihood of survival than others. Our study design enabled us to examine whether and how differences not only in predation risk but also in several abiotic habitat parameters trigger the emergence (or affect the strength and direction) of behavioral syndromes within and among populations. It also allowed investigating the question of whether syndromes would be lost under prolonged absence of environmental triggers, pointing toward a role for behavioral plasticity rather than evolved population differences.

In summary, we predicted that population differences in three personality traits depend on different biotic and abiotic factors (*prediction 1*). As we expect each personality trait to be affected by more than one environmental factor simultaneously, specific one‐dimensional predictions based on recent studies on other organisms could not be formulated. Referring to our second research question, we predicted groups of fish that were maintained under uniform and benign environmental conditions in the laboratory to show homogenization of mean behavioral tendencies compared with the respective wild‐caught cohort (*prediction 2*). Finally, we predicted behavioral syndrome structures to differ in both strength and direction between populations (*prediction 3a*), while differences might disappear after laboratory‐maintenance (*prediction 3b*). Previous studies exhibited an increase in the strength of syndrome structures with increasing predation pressure (Bell, 2005; Bell & Sih, 2007; Dingemanse et al., 2007). However, other environmental factors could alter predation‐dependent syndrome structures, for example, if high predation pressure favors individuals that are active (Archard & Braithwaite, 2011) and show a high shoaling tendency (Godin, 1986), while low transparency of water (low visibility) leads to decreased shoaling behavior (Kelley et al., 2012).

## 2 MATERIALS AND METHODS

### 2.1 Study organism and sampling sites

**Figure 1:**
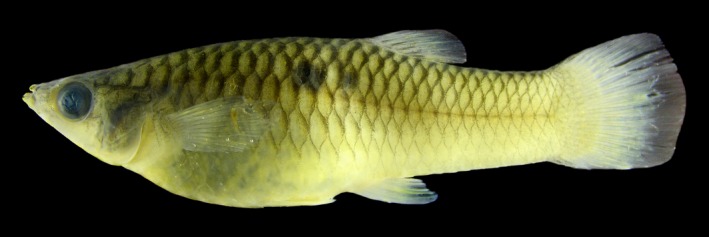
Female peacock molly (*Poecilia vivipara*) with a standard length of 47.5 mm. Courtesy: F. Di Dario

Peacock mollies (*Poecilia vivipara* Bloch & Schneider 1801; Figure 1) have a wide distribution range along the eastern coast of South America, from Venezuela and some islands of the Lesser Antilles in the north to the Lagoa dos Patos in south Brazil (Koerber & Litz, 2014; Lucinda, 2003; Poeser, 2003). The species also occurs in several dozen coastal lagoons in northern Rio de Janeiro state in Brazil, where different populations experience pronounced variation in salinity, ranging from oligosaline (0.2 ppt) to hypersaline, that is, more than twice marine salinity (74 ppt; Di Dario et al., 2013; Correia, 2015). Organisms living under such inhospitable conditions are commonly referred to as “extremophiles” and exhibit an array of physiological and behavioral adaptations to cope with the stressors they are exposed to (Laverty & Skadhauge, 2015; Plath, Tobler, & Riesch, 2015). Constant winds on the shallow water bodies determine generally high levels of dissolved oxygen, but water transparency is highly variable among lagoons due to resuspension of sediments, microalgae concentrations, and dissolved organic carbon (Caliman et al., 2010).

In this study, we investigated *P. vivipara* populations from six coastal lagoons in and around Restinga de Jurubatiba National Park that span the observed range of variation in predation risk and abiotic conditions (Table 1; for location coordinates see Di Dario et al., 2013). Abiotic habitat parameters were assessed during field work using a YSI‐85‐hydrometer (salinity and dissolved oxygen). We classified lagoons into two categories of water transparency (“high” and “low” transparency) taking into account chlorophyll *a* concentrations (Fig. S1), depth, and resuspension of sediments (Caliman et al., 2010), as well as visual evaluation of water samples. Lagoons could clearly be assigned to either of the two categories (Table 1).

The degree of predation risk was based on the records of piscivorous fishes in the studied lagoons during the past 20 years (Araújo, Perez, Magazoni, & Petry, 2014; Di Dario et al., 2013; Felice, 2014). While lagoons differed in the number of piscivorous species (Table S2), no reliable information on the relative abundances of these species was available. We, therefore, decided that a classification into two categories (“high” and “low” predation level) was more biologically meaningful than using absolute numbers of piscivorous species as continuous environmental variable. Lagoons in which both nearshore and pelagic main piscine predators (the erythrinids *Hoplias* aff. *malabaricus* and *Hoplerythrinus unitaeniatus*, and the centropomids *Centropomus parallelus* and *C. undecimalis*, respectively) were present, were assigned to the category “high” predation, while lagoons in which only one or none of these predators occurred were classified as “low” predation.

### 2.2 Test subjects

As personality traits in poeciliid fishes may differ between sexes (Bell, 2005; Harris et al., 2010; Plath & Schlupp, 2008; Riesch et al., 2009) and because sex ratios tended to be female‐biased in some lagoons, we focused on female *P. vivipara* only. Field work was conducted in March and April 2014. We successfully tested a total of 178 females (Lagoa Cabiunas: *n *=* *30, Garças: *n *=* *31, Carapebus: *n *=* *27, Imboassica: *n *=* *24, Catingosa: *n *=* *36, Preta: *n *=* *30). Test subjects were caught with seines (3 mm mesh size) and immediately transferred into water‐filled, aerated plastic boxes placed in the shadow. Test fish remained in the boxes for <2 hr before the personality assessment. On the next day, 14–16 hr after the first personality assessment, we conducted a second (identical) personality assessment with the same individuals to test for individual behavioral consistency. Between both assessments, we kept the test fish in individual perforated plastic bottles (3 L). Bottles were fixed on a rope under the water surface in vegetated areas at the respective sampling sites and left undisturbed overnight. Therefore, test subjects were exposed to similar environmental conditions between the two measurements. After the completion of all measurements, all test subjects were measured for body size (standard length, SL) before they were released into their habitat of origin. Fish that were used to compose stimulus shoals (for the assessment of shoaling tendencies, see below) were collected on the day of the experiments in the respective lagoons, held in aerated plastic boxes in the shadow, and were released into their original habitat after the shoaling assessment.

To conduct tests with individuals that had been acclimated to homogeneous laboratory conditions, we recorded water salinity and collected individuals from four of the six lagoons (Cabiunas: *n *=* *21, Garças: *n *=* *10, Catingosa: *n *=* *11, Preta: *n *=* *36) between September and October 2014. We brought the fish in water‐filled, aerated coolers within <1 h to the Aquatic Animal Facility of the Núcleo em Ecologia e Desenvolvimento Sócioambiental de Macaé. We maintained the fish in aerated, aged, filtered, and salt‐corrected (Natural Ocean^™^) tap water in 30‐L aquaria at densities of less than 25 individuals per aquarium, under a 14 hr light: 10 hr dark photoperiod, for 3 months before we conducted personality assessments. In order to standardize the conditions inside the aquaria, we fed all fish twice a day ad libitum with commercial fish food and *Artemia* nauplii, adjusted temperature at 28 ± 0.5°C in all tanks and made sure that dissolved oxygen was high (>8 mg/L) by equipping all tanks with filters and air stones. Salinity levels resembled those of the respective lagoons. Every week, we removed feces from the bottom and replaced 30% of the water volume.

### 2.3 Personality assessments

We conducted personality assessments with wild‐caught fish directly at the respective sampling sites, thus reducing stress related to handling and transport. Tests with fish maintained under common laboratory conditions were conducted using the same approach in the laboratory facilities. We characterized each test subject along three personality axes: *boldness* as latency to emerge from shelter and enter an unknown area (Biro et al., 2010; Brydges et al., 2008; Harris et al., 2010; Wilson & Godin, 2009), *activity* in an open‐field tank (Archard & Braithwaite, 2011; Bierbach, Sommer‐Trembo, Hanisch, Wolf, & Plath, 2015; Moretz et al., 2007), and *shoaling/sociability* as time spent in the vicinity of a shoal (Cote, Fogarty, Weinersmith, Brodin, & Sih, 2010; Dzieweczynski & Crovo, 2011; Timmermann, Schlupp, & Plath, 2004; Ward et al., 2004); all tests were performed consecutively in the same arena to minimize handling stress.

**Figure 2:**
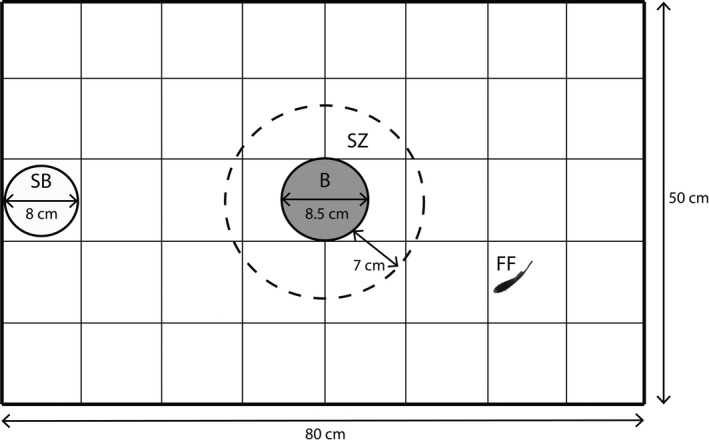
Schematic view of the test tank (view from above). *SB* starting box, a modified plastic yoghurt cup, which served as shelter during the first part of the personality assessment, *B* transparent perforated plastic bottle containing four stimulus fish in the assessment of shoaling/sociability, *SZ* visually marked shoaling zone (shoaling was defined as a focal fish crossing the line at least with its head), *FF* focal female. For display purpose, the focal fish is depicted at an exaggerated size

The test arena consisted of a transparent plastic container (80 × 50 × 50 cm) that was placed on gray cardboard and filled with water from the collection site (wild‐caught fish) or aged filtered, and salt‐corrected tap water (laboratory‐maintained fish) to a height of 15 cm. A grid (10 cm squares) was drawn on the bottom, and all sides were covered with black plastic foil to minimize disturbance. To initiate a trial, we placed the focal individual into a starting box—an opaque 1‐L plastic cup with a diameter of 8 cm that was equipped with a trapdoor (4 × 4 cm)—which we placed at one of the smaller sides of the test arena (Figure 2). We gave the focal female 2 min for acclimation before the trapdoor was remotely opened by a pulley system. We determined the time the focal fish needed to emerge from the starting box (latency time), which is a common measure of boldness in fish (Carter, Feeney, Marshall, Cowlishaw, & Heinsohn, 2013) with bolder fish emerging faster from shelter. We terminated a trial when the female completely emerged from the starting box or after a maximum ceiling value of 10 min (i.e., if the focal fish did not leave the container) and gently moved the fish outside the container with the help of a small aquarium dip net. Afterward, we closed the trapdoor and initiated the second behavioral assessment as soon as the female showed normal swimming behavior (all females resumed swimming after the trapdoor was closed within 2 min). We counted numbers of squares crossed by the focal fish within 5 min, assuming that more active fish would cross more grid squares (*P. reticulata*: Burns, 2008; *P. latipinna*: Muraco, Aspbury, & Gabor, 2014; *P. mexicana*: Bierbach et al., 2015). Directly after the activity assessment, a perforated plastic bottle (diameter: 8.5 cm) containing four conspecific females as a stimulus shoal was placed in the middle of the test arena. Again, we gave the focal female 2 min to habituate to the new situation. During an observation period of 5 min, we determined the time the focal individual spent in a visually marked association zone (7 cm radius around the bottle, equaling about two times the average standard length of the test fish; Figure 2).

### 2.4 Statistical analyses

#### 2.4.1 Effects of environmental factors on personality traits in wild‐caught fish

Our first question was whether the three personality traits were influenced by the different biotic and abiotic environmental factors. First, we calculated intraclass correlation coefficients (ICCs) for each of the three personality traits across all lagoons to estimate the relative variation in behavioral tendencies among versus within lagoons. Using the cohort of wild‐caught fish, we found high ICCs (see section 2.1) for all personality traits, suggesting pronounced differences among lagoons, which could be due to habitat‐specific differences in ecological conditions.

To identify what environmental parameters potentially shape behavioral differences among populations, we conducted three separate generalized linear models (GLMMs) in which one of the three personality traits (mean values across both personality assessments) served as dependent variable, respectively. Again this analysis used the cohort of wild‐caught fish only. The decision to analyze both cohorts of test fish separately was made because a preliminary analysis combining both cohorts found strong effects of rearing conditions (wild‐caught vs. laboratory‐maintained) on two of the three personality traits (Table S3). We specified γ‐shaped distributions for “emergence times” and “shoaling behavior” (each with a log‐link function), whereas a linear distribution was applied for “activity.” We included “predation” and “water transparency” (in both cases categorized as “low” and “high”) as fixed factors and “salinity” and “dissolved oxygen” (DO) as covariates. Due to the limited sample size, we could not include interaction terms.

Note that, for a more intuitive interpretation of the data, we depict “boldness” (maximum emergence time of 600 s—observed individual emergence time) in all figures and discuss this variable in the main text, while unmodified “emergence times” were used in all statistical models.

#### 2.4.2 Homogenization of population differences after laboratory‐maintenance

We asked whether population differences in mean boldness, activity, and shoaling were present also in individuals that had experienced identical conditions (no predation, and uniformly “benign” abiotic conditions except for salinity differences). We thus compared wild‐caught and laboratory‐maintained individuals from four of the six populations. In a first step, we ran a GLMM for each of the three personality traits of the laboratory cohort (similar to the GLMMs for the wild‐caught cohort, see above). For “activity” and “shoaling,” we specified a γ‐shaped distribution with log‐link function. The distribution of “emergence times”, however, showed three peaks, and accordingly, we categorized the data as belonging to one of the following three categories: emergence times between (1) 0–200 s, (2) 201–400 s, and (3) 401–600 s, after which we specified a multinominal distribution function. Factors and covariates were principally the same as described above, but we had to reduce the number of independent variables from four to three due to the smaller sample size in one of the laboratory‐maintained groups (*n *=* *10). For each GLMM, we thus excluded the factor (or covariate) with the weakest effect in the respective GLMM using data from the cohort of wild‐caught fish (see Table 2).

**Table 2:** Results of GLMMs examining the effect of different biotic and abiotic factors (see Table 1) on emergence times (our measure of boldness), activity, and shoaling behavior (sociability) of (*a*) wild‐caught and (*b*) laboratory‐maintained female *P. vivipara*. Significant effects are highlighted in bold font

Factor	Emergence time		Activity		Shoaling	
	χ^2^	*p*	χ^2^	*p*	χ^2^	*p*
(*a*) Wild‐caught cohort						
Predation	**5.10**	**.024**	**9.21**	**.002**	0.51	.47
Turbidity	**6.51**	**.011**	**36.29**	**<.001**	**50.81**	**<.001**
Salinity	0.71	.40	2.65	.10	0.12	.73
DO	0.03	.87	**6.14**	**.013**	0.79	.38
(*b*) Laboratory‐maintained cohort						
Predation	1.46	.23	**6.42**	**.011**	2.63	.11
Turbidity	0.48	.49	1.38	.24	0.20	.65
Salinity	0.55	.46	—	—	—	—
DO	—	—	0.12	.73	0.01	.91

Due to the limited sample size within the laboratory‐maintained cohort, we reduced the number of factors to three, thus avoiding potential overfitting of the models; missing values are indicated by “—.”

In a second step, we ran two principal component analyses (PCA; one for each cohort) on the three personality traits. Both PCAs retrieved one PC with an eigenvalue >1 (in both cases, PC1 explained >60% of the variance; for axis loadings, see Table 3). We plotted those PC scores (mean ± SE) of the four populations for the wild‐caught and laboratory‐maintained cohorts separately to visualize homogenization of behavioral differences after laboratory‐maintenance. Additionally, we calculated ICC values for each personality trait in which we compared wild‐caught and laboratory‐maintained cohorts of the same lagoon (i.e., mean values for each lagoon).

**Table 3:** Results of both PCAs (for the cohorts of wild‐caught and laboratory‐maintained fish, separately) showing axis loadings of the first principal component

Factor	Wild‐caught	Laboratory‐maintained
Emergence time	−0.738	−0.753
Activity	0.868	0.416
Shoaling	0.729	0.788

#### 2.4.3 Behavioral consistency

Consistency of repeatedly measured (behavioral) traits is typically assessed in the form of repeatability (*R*) values, where *R* is defined as *variance among individuals/*(*variance among individuals + variance within individuals*) (Bell et al., 2009). To obtain variance parameters from both datasets collected for this study, we used univariate mixed models for each behavioral trait and for wild‐caught and laboratory‐maintained fish separately (Nakagawa & Schielzeth, 2010). We used original data of both personality assessments as dependent variable and included a repeated measures factor. We included “fish ID” as random factor and “lagoon” as fixed factor in all models. Significant deviations of *R* from zero were tested with likelihood ratio tests.

#### 2.4.4 Behavioral syndrome structure

To test for potential differences among populations in the strength and direction of behavioral syndromes, we initially intended to calculate multivariate mixed models including all three personality traits. Multivariate mixed models provide the possibility to split phenotypic correlations into correlations on the among‐individual level and the residual covariance level, respectively, which allows a more accurate calculation of behavioral syndrome structures (Brommer, 2013; Dingemanse & Dochtermann, 2013; Dingemanse, Dochtermann, & Nakagawa, 2012). However, due to the widely differing distribution patterns of our measures of boldness, activity, and shoaling, it was not possible to integrate all three personality traits in one model. We, therefore, decided to use a more conservative approach, which does not control for possible overestimations of syndrome structures through “individual gambit” (Brommer, 2013), but merely allowed us to uncover behavioral correlations on the phenotypic level. We ran Spearman rank correlations on individual values of boldness, activity, and shoaling within each lagoon (and for wild‐caught and laboratory‐maintained individuals, respectively). We corrected α‐levels for multiple testing as α’ = 0.05/3 = 0.017.

Moreover, we asked whether and how environmental factors affect the overall strength of behavioral syndromes. Therefore, we calculated cumulative syndrome strengths for each population (for wild‐caught and laboratory‐maintained individuals, separately) by summing all pairwise Spearman rank correlation coefficients (absolute, sign‐free values) for all three personality traits. We used the resulting values as dependent variable in a GLM and included the aforementioned factors and covariates.

All statistical tests were conducted using IBM SPSS 23.0.

## 3 RESULTS

### 3.1 Ambient environmental conditions drive population differences in personality traits

We first analyzed the cohort of wild‐caught individuals from the six lagoons. ICC analyses indicated strong consistency in personality traits among individuals within lagoons (boldness: ICC* *=* *0.698, *p *=* *.007; activity: ICC* *=* *0.928, *p *<* *.001; shoaling: ICC* *=* *0.961, *p *<* *.001). In addition, these results suggest consistent differences in mean behavioral tendencies between lagoons.

In a second step, we tested whether environmental parameters shape the uncovered personality differences among lagoons. In line with *prediction 1*, generalized linear models (GLMMs) for each of the three personality traits found emergence times to be significantly influenced by “predation” and “water transparency” (Table 2a), with emergence times being higher under high predation threat (estimated marginal means, EMMs ± SE, low predation: 54.89 ± 14.17 s, high predation: 143.52 ± 34.47 s) and under low water transparency (high water transparency: 59.49 ± 9.30 s, low water transparency: 132.43 ± 32.02 s). Activity was affected by “predation,” “water transparency,” and ambient oxygen concentrations (“DO”; Table 2a). Activity decreased with high levels of predation (EMMs, low predation: 78.38 ± 5.73 squares, high predation: 49.18 ± 5.48 squares), under low water transparency conditions (high water transparency: 83.95 ± 3.43 squares, low water transparency: 43.61 ± 5.22 squares), and with increasing DO (post‐hoc Spearman rank correlation: *r *=* *−.15, *p *=* *.066). Shoaling behavior was significantly influenced by “water transparency” (Table 2a), with lower shoaling times under low water transparency conditions (EMMs, high water transparency: 180.95 ± 20.38 s, low water transparency: 37.64 ± 6.45 s). Note that “salinity” affected none of the personality traits (Table 2a).

### 3.2 Homogenization of population differences after laboratory‐maintenance

In accordance with *prediction 2*, the results of our GLMMs using data from the four groups of laboratory‐maintained individuals indicate pronounced shifts in mean behavioral tendencies in this cohort such that most effects observed in the analysis of wild‐caught individuals could not be detected (Table 2a, b). Only activity was significantly influenced by the level of predation that the fish had experienced in their natural habitats (Table 2b). Likewise, ICC values (comparing wild‐caught and laboratory cohorts of the same lagoon, respectively) of boldness and shoaling tendency were low and nonsignificant (boldness: ICC* *=* *−0.002, *p *=* *.51; shoaling: ICC* *=* *0.421, *p *=* *.33), whereas activity had a higher, albeit nonsignificant ICC value (ICC* *=* *0.609, *p *=* *.17).

**Figure 3:**
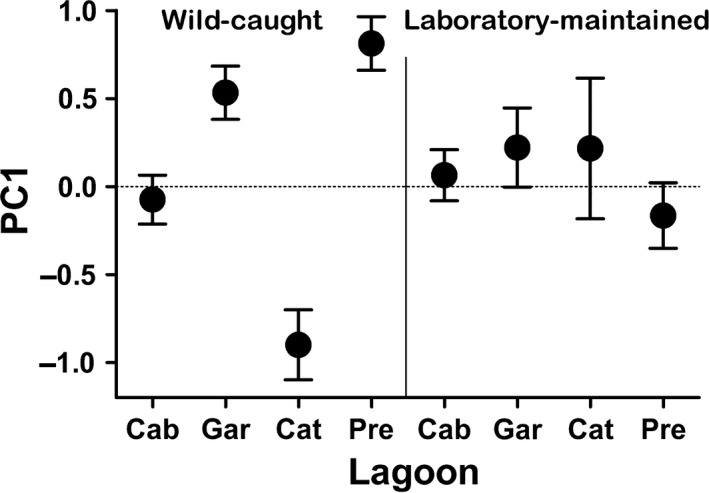
Visualization of behavioral homogenization after maintenance in the laboratory. Principal component scores (PC1, mean ± *SE*) are shown for each of the following lagoons: *Cab *=* *Cabiunas, *Gar *=* *Garças, *Cat *=* *Catingosa, *Pre *=* *Preta

In support of these results, visual inspection of PC scores of different populations suggests homogenization of behavioral tendencies in all laboratory‐maintained groups in a way that their mean PC scores were intermediate to the variation seen in wild‐caught fish (Figure 3). Median values of the raw data for all three personality traits and both cohorts of test fish are depicted in Fig. S4.

### 3.3 Behavioral consistency

In the wild‐caught cohort, we found shoaling tendencies to be repeatable (*R *=* *0.19, *p *=* *.013), while consistency in boldness was slightly lower and not statistically significant (*R *=* *0.13, *p *=* *.075). For activity, the among‐individual variance estimate was close to zero, which resulted in a nonsignificant *R*‐value of 0. For all personality traits, we found a significant influence of the fixed factor “lagoon” (boldness: *F*_5,137_ **=* *3.26, *p *=* *.008; activity: *F*_5,280_ **=* *13.03, *p *<* *.001; shoaling: *F*_5,137_ **=* *24.80, *p *<* *.001), suggesting differences in consistency among populations.

When considering the laboratory‐reared cohort, we found all three personality traits to be highly repeatable (boldness: *R *=* *0.37, *p *<* *.001; activity: *R *=* *0.57, *p *<* *.001; shoaling: *R *=* *0.28, *p *=* *.006). The factor “lagoon” did not affect any of the personality traits (boldness: *F*_3,74_ **=* *1.59, *p *=* *.20; activity: *F*_3,74_ **=* *2.71, *p *=* *.51; shoaling: *F*_3,74_ **=* *2.24, *p *=* *.091; for a brief discussion of cohortwise differences in behavioral consistency, see Supporting information S5).

### 3.4 Behavioral syndrome structures

**Figure 4:**
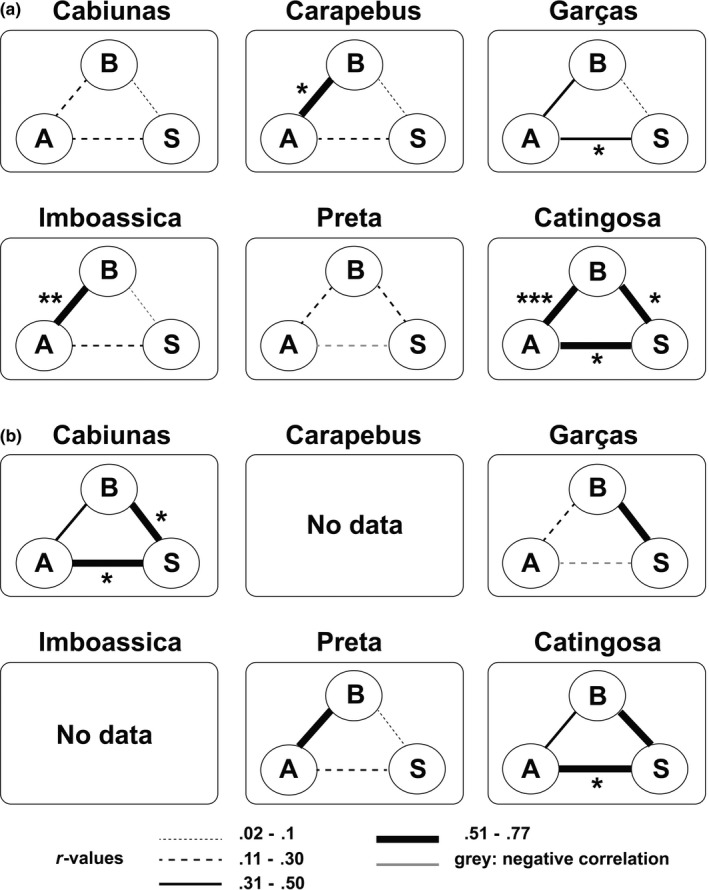
(a) Syndrome structures between boldness (*B*), activity (*A*), and shoaling behavior (*S*) in six *P. vivipara* populations (wild‐caught fish). Connecting lines between the three personality traits represent the strength of the correlations, estimated via Spearman rank correlation coefficients (*r*). (b) Syndrome structure in four populations from which focal individuals had been maintained in the laboratory before testing

In accordance with *prediction 3a*, visual evaluation suggests that behavioral syndrome structures vary substantially in their strength and direction among lagoons of the wild‐caught cohort (Figure 4a). Interestingly, neither visual evaluation of syndrome structures (Figure 4a) nor our GLM using cumulative correlation coefficients per population (only wild‐caught cohort) detected any effects of predation level (nor any other environmental parameter) on the overall strength of behavioral syndromes (GLM: *F *<* *0.63, *p *>* *.56, *n *=* *6).

Considering wild‐caught fish, no significant correlations between behavioral traits were found in the Cabiunas (*r *<* *.27, *p *>* *.20, *n *=* *25) and Preta populations (*r *<* *.24, *p *>* *.27, *n *=* *24; Figure 4a). In half of the lagoons, we found a significant positive correlation between boldness and activity (*r *>* *.55, *p *<* *.005). The tightest syndrome structure between all three behavioral traits was found in the Catingosa population (*r *>* *.50, *p *<* *.015, *n *=* *23; Figure 4a), one of the two populations that showed high behavioral consistency (see above).

Syndrome structures of laboratory‐maintained groups changed unpredictably in direction and/or strength compared with the corresponding wild‐caught group of individuals (Figure 4b). Contrary to *prediction 3b*, the overall strength of syndrome structures did not decrease after laboratory‐maintenance.

## 4 DISCUSSION

### 4.1 Effects of environmental factors on personality traits

We compared three personality traits (boldness, activity, and sociability/shoaling behavior) of female *P. vivipara* from six coastal lagoons (populations) that differed markedly in several biotic and abiotic habitat parameters. In accordance with *prediction 1*, we found pronounced population differences in all personality traits that could be related to different environmental parameters.

Interestingly, water transparency was the factor with the strongest influence on all three personality traits. Shoaling tendencies were considerably lower in populations living under low water transparency conditions compared with populations from lagoons with clear water. In support of this finding, studies on other freshwater fishes also reported on shoals being less cohesive under turbid water conditions (*P. reticulata*: Kimbell & Morrell, 2015; *Melanotaenia australis*: Kelley et al., 2012). One explanation for this effect is that predators that rely on visual prey detection face difficulties in targeting their prey at greater distance under decreased water transparency, which in turn can decrease effective predation pressure, especially for small prey species (reviewed in Utne‐Palm, 2002). Following this line of argumentation, prey species are expected to shoal less under low water transparency conditions because the costs of living in a shoal (e.g., competition for resources) outweigh the benefits arising from protection from visually orientated predators (Pitcher & Parrish, 1993). An alternative, not mutually exclusive explanation was provided by Kimbell and Morrell (2015) who observed that guppies under turbid water conditions not only shoaled less but also increased their freezing behavior after a predator attack. Freezing represents alternative predator‐avoidance behavior (Brown & Godin, 1999) and is sometimes also used as a measure of boldness (Bierbach et al., 2015; Piyapong et al., 2010). The authors argue that, due to the reduction/loss of visual contact among prey fish, individuals under turbid conditions are forced to rely more on individual antipredator behavior rather than forming shoals.

A combination of both hypotheses likely explains the findings of our present study: We found *P. vivipara* from lagoons with low water transparency to be shyer and less active, which could indeed reflect an overall more cautious (individual) behavioral coping style caused by the limited visual contact between shoal members (Kimbell and Morrell 2015). On the other hand, the first hypothesis explains decreased shoaling behavior as a consequence of relaxed effective predation pressure (i.e., independent of actual densities of co‐occurring predatory species). Following this idea, we would not necessarily expect a main effect of the factor “predation pressure” on shoaling tendencies in habitats with low visibility, which was confirmed by the results of our study (note that, due to statistical limitations, we could not test for an interaction effect between “predation” and “water transparency,” and studies comparing a larger number of populations will be needed to test for such an effect). Populations under high predation pressure were, however, shyer and less active, but these effects were weaker than those explained by the factor “water transparency” (Table 2a).

Activity was also affected by DO in a way that fish were more active under lower oxygen concentrations. In theory, one would expect fish to be less (not more) active under low‐oxygen conditions (Schurmann & Steffensen, 1994) because more energy must be allocated to gill ventilation (Petrosky & Magnuson, 1973), thereby increasing total energy expenditure. Thus, energetically costly behaviors like courtship/reproductive behavior, feeding, and rapid swimming (e.g., escape from predators) are reduced (Hubbs, Baird, & Gerald, 1967) or replaced by less energy‐demanding behaviors (Whoriskey, Gaudreault, Martel, Campeau, & FitzGerald, 1985) under low‐oxygen conditions, such as hypoxia (reviewed in Kramer, 1987). However, in our study, differences in DO among the different lagoons were relatively small and DO levels were generally within the range of well‐oxygenated water. While we have no obvious explanation at hand for the negative correlation between DO and activity, we tentatively argue that other environmental factors, which have not been assessed in our present study, might be intercorrelated with the factor “DO.” One possible scenario is that slightly lower DO indicates that densities of (oxygen‐producing) microalgae are also low. Microalgae serve as a food source for several poeciliids (Dussault & Kramer, 1981; Karino & Haijima, 2004; Meffe & Snelson, 1989) including members of the subgenus *Mollienesia* to which *P. vivipara* belong (Scharnweber, Plath, & Tobler, 2011b; Scharnweber, Plath, Winemiller, & Tobler, 2011a), and fish from habitats with low algal productivity might need to swim more actively between food patches to find sufficient food.

In summary, our study highlights the importance of evaluating multiple rather than single environmental variables in studies of phenotypic divergence in natural populations. Natural environments are highly complex, and so it is to be expected that different selective agents can act in concert but also in opposition when exerting selection on organismal phenotypes (e.g., Langerhans & Riesch, 2013). Furthermore, we showed that abiotic factors (especially water transparency) can have strong effects on personality traits and should therefore be given more attention in future research on animal personality.

### 4.2 Homogenization of population differences after laboratory‐maintenance

A multitude of studies reported on differences in personality traits like boldness, exploration, activity, aggressiveness, or shoaling among fish populations that are exposed to varying environmental conditions (Seghers, 1974; Magurran, 1986; Fraser & Gilliam, 1987; Brown et al., 2005; Alvarés & Bell, 2007; Harris et al., 2010; Archard & Braithwaite, 2011; Borner et al., 2015). However, there is only limited information about the relative contributions of heritable (genetic) versus plastic components to these population differences (Bell, 2005; Brown, Burgess, & Braithwaite, 2007; Riesch et al., 2009). To investigate the degree of plasticity of mean behavioral traits among populations, we compared the behavior of wild‐caught test subjects and fish from the same lagoons that had been maintained in the laboratory under uniform (thus homogenized, except for salinity differences) environmental conditions for at least 3 months. In accordance with *prediction 2*, we found homogenization of mean behavioral tendencies, such that differences that became apparent among wild‐caught populations were almost entirely absent in fish that had been kept in the laboratory. The sole exception was the effect of predation pressure on swimming activity, which remained statistically significant in laboratory‐maintained fish, even though the effect strength was lower than in the wild‐caught cohort. This could either indicate a long‐lasting experiential or a heritable effect (Dingemanse et al., 2009; van Oers, de Jong, van Noordwijk, Kempenaers, & Drent, 2005). However, the question remains why only the specific effect of predation pressure on swimming activity persisted, whereas neither the effect of predation pressure on boldness nor an effect of any other environmental factor on activity was retained in the laboratory‐maintained cohort. We hypothesize that this could be the result of correlated evolution of swimming activity with another trait we did not quantify in the present study (Losos, 2011). For example, a recent study on correlated evolution of certain behavioral and morphological phenotypes in zebrafish (*Danio rerio*), might provide a potential explanation for this intriguing pattern (Kern, Robinson, Gass, Godwin, & Langerhans, 2016). In that study, artificial selection for boldness also leads to corresponding morphological changes usually found in high‐predation environments (i.e., larger caudal peduncle area and increased fast‐start response). A similar phenomenon (but in reverse) might explain the persistence of lower activity in fish from high‐predation environments in our study, because “high‐predation body shapes” have been demonstrated to be heritable for several generations in other poeciliid fishes (e.g., Langerhans, 2009; Langerhans, Layman, Shokrollahi, & DeWitt, 2004), and will therefore not have changed after only 3 months under common‐garden conditions. As previous studies reported on similar high‐ and low‐predation body shapes also in *P. vivipara* from the lagoons evaluated here and in their vicinity (Araújo et al., 2014; Gomes & Monteiro, 2008), it is possible that the persistence of lower activity after the laboratory‐maintenance phase is simply indicative of the persistence of high‐predation and low‐predation body shapes in our test fish. However, we are aware that this explanation rests on the assumption that altered body shape only corresponds with differences in activity in our system, but not in traits like boldness and shoaling—an assumption that is currently not supported by empirical data. Future studies should investigate the potential for such correlated evolution of behaviors and other traits (including body shape) in *P. vivipara* further.

Another potential reason for the overall low persistence of personality traits in this particular system is the high degree of seasonal and yearly variation in some of the habitat characteristics. Chagas and Suzuki (2005) reported on strong seasonal variation in parameters like DO and salinity in one lagoon to the east of our study area. Furthermore, our study system undergoes cyclical changes by flooding every few decades, and catastrophic desiccation of the brackish to saltwater lagoons might also occur (Almeida, 2013; Felice, 2014; de Macedo‐Soares, Petry, Farjalla, & Caramaschi, 2010). Hence, recurrent fluctuation in various abiotic and biotic factors drives phenotypic diversification on a small geographic scale but potentially also selects for plasticity rather than heritability.

Nonetheless, our results suggest that, in general, personality traits in our study species have a strong plastic component and can be altered by immediate experience. This finding is congruent with studies on other fish species that found individuals to change their personality traits in response to altered environmental or social conditions (*Onchorhynchus mykiss*: Frost, Winrow‐Giffen, Ashley, & Sneddon, 2007; *Pomacentrus moluccensis*: Biro, Beckmann & Stamps 2008; *P. mexicana*: C. Sommer‐Trembo et al. unpublished).

### 4.3 Behavioral syndrome structures

Correlations between two or more personality traits (behavioral syndromes) have been observed in a variety of fishes (e.g., *Amatitlania siquia*: Mazué et al., 2015; *D. rerio*: Moretz et al., 2007; *G. aculeatus*: Ward et al., 2004; Bell, 2005; Dingemanse et al., 2007; *Lepomis macrochirus*: Wilson & Godin, 2009; *P. mexicana*: Bierbach et al., 2015), while the strength and direction of these correlations may vary between populations of the same species (Bell, 2005; Dingemanse et al., 2007). One explanation for the existence of behavioral syndromes is given by the “adaptive hypothesis,” which assumes selection to favor distinct combinations of behavioral traits (correlational selection) dependent on ambient environmental factors. Differences in predation regimes are assumed to be one key selective agent to shape population differences in syndrome structures (Bell & Sih, 2007). In the present study, syndrome structures differed widely among populations, both in their strength and in direction (*prediction 3a*). However, correlational selection is unlikely to explain our findings because syndrome structures varied markedly between wild‐caught and laboratory‐maintained fish of the same lagoon. It has to be mentioned though that we could not test for possible correlations on the residual level which could have led to an exaggeration/bias in our estimates of syndrome structures (Brommer, 2013; Dingemanse & Dochtermann, 2013) and so further studies will be needed to rule out the possibility that correlational selection is acting to shape behavioral syndromes in this study system. Furthermore, the additive strength of syndrome structure could not be linked to any particular ecological factor (including predator regime, negating *prediction 3b*). Given that different environmental factors (including additional environmental factors not evaluated in this study as well as combinations and interactions of all factors) simultaneously affected single personality traits in different directions, it is not surprising that correlations between these traits varied unpredictably between populations. However, our sample size was restricted to six populations and future studies with a larger sample size are desirable to identify under which environmental conditions different behavioral traits might be selected for in a correlated fashion and under which environmental conditions they might not.

## DATA ACCESSIBILITY

Datasets supporting our findings can be found as additional spreadsheet files.

## CONFLICT OF INTEREST

None declared.

## AUTHORS’ CONTRIBUTIONS

MP and CST conceived the ideas and designed methodology; AP, GGS, SV, JG, JB, SK, and JAC collected the data; CST analyzed the data; CST wrote the first draft of the manuscript. CST, MP, and RR led the further writing of the manuscript. All authors contributed critically to the drafts and gave final approval for publication.

## References

[ece33165-bib-0001] Almeida, A. M. V. (2013). Carapebus: nas páginas do passado, (309 p). Niterói, Brazil: Ed. Muiraquitá.

[ece33165-bib-0002] Álvarez, D. , & Bell, A. M. (2007). Sticklebacks from streams are more bold than sticklebacks from ponds. Behavioural Processes, 76, 215–217. 17583445 10.1016/j.beproc.2007.05.004

[ece33165-bib-0003] Araújo, M. S. , Perez, S. I. , Magazoni, M. J. C. , & Petry, A. C. (2014). Body size and allometric shape variation in the molly *Poecilia vivipara* along a gradient of salinity and predation. BMC Evolutionary Biology, 14, 251. 25471469 10.1186/s12862-014-0251-7 PMC4272540

[ece33165-bib-0004] Archard, G. A. , & Braithwaite, V. A. (2011). Increased exposure to predators increases both exploration and activity level in *Brachyrhaphis episcopi* . Journal of Fish Biology, 78, 593–601. 21284637 10.1111/j.1095-8649.2010.02880.x

[ece33165-bib-0005] Bell, A. M. (2005). Behavioural differences between individuals and two populations of stickleback (*Gasterosteus aculeatus*). Journal of Evolutionary Biology, 18, 464–473. 15715852 10.1111/j.1420-9101.2004.00817.x

[ece33165-bib-0006] Bell, A. M. (2007). Future directions in behavioural syndromes research. Proceedings of the Royal Society B, 274, 755–761. 17251088 10.1098/rspb.2006.0199 PMC1919401

[ece33165-bib-0007] Bell, A. M. , Hankison, S. J. , & Laskowski, K. L. (2009). The repeatability of behaviour: A meta‐analysis. Animal Behaviour, 77, 771–783. 24707058 10.1016/j.anbehav.2008.12.022 PMC3972767

[ece33165-bib-0008] Bell, A. M. , & Sih, A. (2007). Exposure to predation generates personality in threespined sticklebacks (*Gasterosteus aculeatus*). Ecology Letters, 10, 828–834. 17663716 10.1111/j.1461-0248.2007.01081.x

[ece33165-bib-0009] Bierbach, D. , Sommer‐Trembo, C. , Hanisch, J. , Wolf, M. , & Plath, M. (2015). Personality affects mate choice: bolder males show stronger audience effects under high competition. Behavioral Ecology, 26, 1314–1325.

[ece33165-bib-0010] Biro, P. A. , Beckmann, C. , & Stamps, J. A. (2010). Small within‐day increases in temperature affects boldness and alters personality in coral reef fish. Proceedings of the Royal Society B, 277, 71–77. 19793748 10.1098/rspb.2009.1346 PMC2842624

[ece33165-bib-0011] Borner, K. K. , Krause, S. , Mehner, T. , Uusi‐Heikkilä, S. , Ramnarine, I. W. , & Krause, J. (2015). Turbidity affects social dynamics in Trinidadian guppies. Behavioral Ecology and Sociobiology, 69, 645–651.

[ece33165-bib-0012] Brommer, J. E. (2013). On between‐individual and residual (co)variances in the study of animal personality: Are you willing to take the ‘individual gambit’? Behavioral Ecology and Sociobiology, 67, 1027–1032.

[ece33165-bib-0013] Brown, C. , Burgess, F. , & Braithwaite, V. A. (2007). Heritable and experiential effects on boldness in a tropical poeciliid. Behavioral Ecology and Sociobiology, 62, 237–243.

[ece33165-bib-0014] Brown, G. E. , & Godin, J.‐G. J. (1999). Chemical alarm signals in wild Trinidadian guppies (*Poecilia reticulata*). Canadian Journal of Zoology, 77, 562–570.

[ece33165-bib-0015] Brown, C. , Jones, F. , & Braithwaite, V. (2005). In situ examination of boldness‐shyness traits in the tropical poeciliid, *Brachyraphis episcopi* . Animal Behaviour, 70, 1003–1009.

[ece33165-bib-0016] Brydges, N. M. , Colegrave, N. , Heathcote, R. J. P. , & Braithwaite, V. A. (2008). Habitat stability and predation pressure affect temperament behaviours in populations of three‐spined sticklebacks. Journal of Animal Ecology, 77, 229–235. 18217944 10.1111/j.1365-2656.2007.01343.x

[ece33165-bib-0017] Burns, J. G. (2008). The validity of three tests of temperament in guppies (*Poecilia reticulata*). Journal of Comparative Psychology, 122, 344–356. 19014258 10.1037/0735-7036.122.4.344

[ece33165-bib-0018] Caliman, A. , Carneiro, L. S. , Santangelo, J. M. , Guariento, R. D. , Pires, A. P. F. , Suhett, A. L. , … Farjalla, V. F. (2010). Temporal coherence among tropical coastal lagoons: A search for patterns and mechanisms. Brazilian Journal of Biology, 70, 803–814. 10.1590/s1519-69842010000400011 21085785

[ece33165-bib-0019] Carter, A. J. , Feeney, W. E. , Marshall, H. H. , Cowlishaw, G. , & Heinsohn, R. (2013). Animal personality: What are behavioural ecologists measuring? Biological Reviews, 88, 465–475. 23253069 10.1111/brv.12007

[ece33165-bib-0020] Chagas, G. G. , & Suzuki, M. S. (2005). Seasonal hydrochemical variation in a tropical coastal lagoon (Açu Lagoon, Brazil). Brazilian Journal of Biology, 65, 597–607. 10.1590/s1519-69842005000400006 16532183

[ece33165-bib-0021] Correia, L. V. (2015). O efeito da salinidade em Poecilia vivipara Bloch & Schneider 1801 (Poeciliidae): explorando as variações no provisionamento materno e outras táticas reprodutivas. Dissertação de Mestrado em Ecologia. Rio de Janeiro: Universidade Federal do Rio de Janeiro: UFRJ, 93p.

[ece33165-bib-0022] Cote, J. , Fogarty, S. , Weinersmith, K. , Brodin, T. , & Sih, A. (2010). Personality traits and dispersal tendency in the invasive mosquitofish (*Gambusia affinis*). Proceedings of the Royal Society B, 277, 1571–1579. 20071380 10.1098/rspb.2009.2128 PMC2871838

[ece33165-bib-0023] Di Dario, F. , Petry, A. C. , de Souza Pereira, M. M. , Mincarone, M. M. , Soares Agostinho, L. , Martins Camara, E. , … de Britto, M. R. (2013). An update on the fish composition (Teleostei) of the coastal lagoons of the Restinga de Jurubatiba National Park and the Imboassica Lagoon, northern Rio de Janeiro State. Acta Limnologica Brasiliensia, 25, 257–278.

[ece33165-bib-0024] Dingemanse, N. J. , & Dochtermann, N. A. (2013). Quantifying individual variation in behaviour: Mixed‐effect modelling approaches. Behavioral Ecology and Sociobiology, 82, 39–54. 10.1111/1365-2656.12013 23171297

[ece33165-bib-0025] Dingemanse, N. J. , Dochtermann, N. A. , & Nakagawa, S. (2012). Defining behavioural syndromes and the role of ‘syndrome deviation’ in understanding their evolution. Behavioral Ecology and Sociobiology, 66, 1543–1548.

[ece33165-bib-0026] Dingemanse, N. J. , Kazem, A. J. N. , Réale, D. , & Wright, J. (2010). Behavioural reaction norms: Animal personality meets individual plasticity. Trends in Ecology and Evolution, 25, 81–89. 19748700 10.1016/j.tree.2009.07.013

[ece33165-bib-0027] Dingemanse, N. J. , van der Plas, F. , Wright, J. , Réale, D. , Schrama, M. , Roff, D. A. , … Barber, I. (2009). Individual experience and evolutionary history of predation affect expression of heritable variation in fish personality and morphology. Proceedings of the Royal Society B, 276, 1285–1293. 19129142 10.1098/rspb.2008.1555 PMC2660958

[ece33165-bib-0028] Dingemanse, N. J. , Wright, J. , Kazem, A. J. , Thomas, D. K. , Hickling, R. , & Dawnay, N. (2007). Behavioural syndromes differ predictably between 12 populations of three‐spined stickleback. Journal of Animal Ecology, 76, 1128–1138. 17922709 10.1111/j.1365-2656.2007.01284.x

[ece33165-bib-0029] Dussault, G. V. , & Kramer, D. L. (1981). Food and feeding behavior of the guppy, *Poecilia reticulata* (Pisces: Poeciliidae). Canadian Journal of Zoology, 59, 684–701.

[ece33165-bib-0030] Dzieweczynski, T. L. , & Crovo, J. A. (2011). Shyness and boldness differences across contexts in juvenile three‐spined stickleback *Gasterosteus aculeatus* from an anadromous population. Journal of Fish Biology, 79, 776–788. 21884112 10.1111/j.1095-8649.2011.03064.x

[ece33165-bib-0031] Felice, B. C. (2014) Dinâmica de Metacommunidades de peixes em ecossistemas costeiros: uma abordagem com lagoas e poças do Parque Nacional da Restinga de Jurubatiba. Dissertação de Mestrado em Ciências Ambientais e Conservação. Universidade Federal do Rio de Janeiro campus Macaé Rio de Janeiro: UFRJ, 122 p.

[ece33165-bib-0032] Fraser, D. F. , & Gilliam, J. F. (1987). Feeding under predation hazard: Response of the guppy and Hart's rivulus from sites with contrasting predation hazard. Behavioral Ecology and Sociobiology, 21, 203–209.

[ece33165-bib-0033] Freund, J. , Brandmaier, A. M. , Lewejohann, L. , Kirste, I. , Kritzler, M. , Krüger, A. , … Kempermann, G. (2013). Emergence of individuality in genetically identical mice. Science, 340, 756–759. 23661762 10.1126/science.1235294

[ece33165-bib-0034] Frost, A. J. , Winrow‐Giffen, A. , Ashley, P. J. , & Sneddon, L. U. (2007). Plasticity in animal personality traits: Does prior experience alter the degree of boldness? Proceedings of the Royal Society B, 274, 333–339. 17164196 10.1098/rspb.2006.3751 PMC1702388

[ece33165-bib-0035] Godin, J.‐G. J. (1986). Antipredator function of shoaling in teleost fishes: A selective review. Le Naturaliste Canadien, 113, 241–250.

[ece33165-bib-0036] Gomes, J. L. Jr , & Monteiro, L. R. (2008). Morphological divergence patterns among populations of *Poecilia vivipara* (Teleostei Poeciliidae): Test of an ecomorphological paradigm. Biological Journal of the Linnean Society, 93, 799–812.

[ece33165-bib-0037] Gosling, S. D. (2001). From mice to men: What we can learn about personality from animal research. Psychological Bulletin, 127, 45–86. 11271756 10.1037/0033-2909.127.1.45

[ece33165-bib-0038] Gosling, S. D. , & John, O. P. (1999). Personality dimensions in nonhuman animals: A cross‐species review. Current Directions in Psychological Science, 8, 69–75.

[ece33165-bib-0039] Harris, S. , Ramnarine, I. W. , Smith, H. G. , & Pettersson, L. B. (2010). Picking personalities apart: Estimating the influence of predation, sex and body size on boldness in the guppy *Poecilia reticulata* . Oikos, 119, 1711–1718.

[ece33165-bib-0040] Hubbs, C. , Baird, R. C. , & Gerald, J. W. (1967). Effects of dissolved oxygen concentration and light intensity on activity cycles of fishes inhabiting warm springs. American Midland Naturalist, 77, 104–115.

[ece33165-bib-0041] Karino, K. , & Haijima, Y. (2004). Algal‐diet enhances sexual ornament, growth and reproduction in the guppy. Behaviour, 141, 585–601.

[ece33165-bib-0042] Kelley, J. L. , Phillips, B. , Cummins, G. H. , & Shand, J. (2012). Changes in the visual environment affect colour signal brightness and shoaling behaviour in a freshwater fish. Animal Behaviour, 83, 783–791.

[ece33165-bib-0043] Kern, E. M. A. , Robinson, D. , Gass, E. , Godwin, J. , & Langerhans, R. B. (2016). Correlated evolution of personality, morphology and performance. Animal Behaviour, 117, 79–86. 10.1016/j.anbehav.2016.04.007 PMC5791543 29398712

[ece33165-bib-0044] Kimbell, H. S. , & Morrell, L. J. (2015). Turbidity influences individual and group level responses to predation in guppies, *Poecilia reticulata* . Animal Behaviour, 103, 179–185.

[ece33165-bib-0045] Kobler, A. , Maes, G. E. , Humblet, Y. , Volckaert, A. M. , & Eens, M. (2011). Temperament traits and microhabitat use in bullhead, *Cottus perifretum*: Fish associated with complex habitats are less aggressive. Behaviour, 148, 603–625.

[ece33165-bib-0046] Koerber, S. , & Litz, T. O. (2014). On the erroneous records of *Poecilia vivipara* from Argentina. Ichthyological Contributions of Peces Criollos, 33, 1–4.

[ece33165-bib-0047] Kramer, D. L. (1987). Dissolved oxygen and fish behavior. Environmental Biology of Fishes, 18, 81–92.

[ece33165-bib-0048] Langerhans, R. B. (2009). Trade‐off between steady and unsteady swimming underlies predator‐driven divergence in *Gambusia affinis* . Journal of Evolutionary Biology, 22, 1057–1075. 21462405 10.1111/j.1420-9101.2009.01716.x

[ece33165-bib-0049] Langerhans, R. B. , Layman, C. A. , Shokrollahi, A. M. , & DeWitt, T. J. (2004). Predator‐driven phenotypic diversification in *Gambusia affinis* . Evolution, 58, 2305–2318. 15562692 10.1111/j.0014-3820.2004.tb01605.x

[ece33165-bib-0050] Langerhans, R. B. , & Riesch, R. (2013). Speciation by selection: A framework for understanding ecology's role in speciation. Current Zoology, 59, 31–52.

[ece33165-bib-0051] Laverty, G. , & Skadhauge, E. (2015). Hypersaline environments In Riesch R., Tobler M., & Plath M. (Eds.), Extremophile fishes. Ecology, evolution, and physiology of Teleosts in extreme environments (pp. 85–106). Heidelberg, New York: Springer.

[ece33165-bib-0052] Losos, J. B. (2011). Convergence, adaptation, and constraint. Evolution, 65, 1827–1840. 21729041 10.1111/j.1558-5646.2011.01289.x

[ece33165-bib-0053] Lucinda, P. H. F. (2003). Family Poeciliidae In Reis R. E., Kullander S. O., & Ferraris C. J. Jr. (Eds.), Check list of the freshwater fishes of South and Central America (pp. 555–581). Porto Alegre, Brazil: Edipucrs.

[ece33165-bib-0054] de Macedo‐Soares, P. H. M. , Petry, A. C. , Farjalla, V. F. , & Caramaschi, E. P. (2010). Hydrological connectivity in coastal inland systems: Lessons from a Neotropical fish metacommunity. Ecology of Freshwater Fish, 19, 7–18.

[ece33165-bib-0055] Magurran, A. E. (1986). Predator inspection behaviour in minnow shoals: Differences between populations and individuals. Behavioral Ecology and Sociobiology, 19, 267–273.

[ece33165-bib-0056] Magurran, A. E. , & Seghers, B. H. (1991). Variation in schooling and aggression amongst guppy (*Poecilia reticulata*) populations in Trinidad. Behaviour, 118, 214–234.

[ece33165-bib-0057] Magurran, A. E. , & Seghers, B. H. (1994). Predator inspection behaviour covaries with schooling tendency amongst wild guppy, *Poecilia reticulata*, populations in Trinidad. Behaviour, 128, 121–134.

[ece33165-bib-0058] Magurran, A. E. , Seghers, B. H. , Carvalho, G. R. , & Shaw, P. W. (1992). Behavioural consequences of an artificial introduction of guppies (*Poecilia reticulata*) in N. Trinidad: Evidence for the evolution of anti‐predator behaviour in the wild. Proceedings of the Royal Society B, 248, 117–122.

[ece33165-bib-0059] Mazué, G. P. F. , Dechaume‐Moncharmont, F. , & Godin, J.‐G. J. (2015). Boldness‐exploration behavioral syndrome: Interfamily variability and repeatability of personality traits in the young of the convict cichlid (*Amatitlania siquia*). Behavioral Ecology, 26, 900–908.

[ece33165-bib-0060] Meffe, G. K. , & Snelson, F. F. (1989). An ecological overview of poeciliid fishes In Meffe G. K., & Snelson F. F. (Eds.), Ecology and evolution of livebearing fishes Poeciliidae (p. 7). Englewood Cliffs: Prentice Hall.

[ece33165-bib-0061] Moretz, J. A. , Martins, E. P. , & Robison, B. D. (2007). Behavioral syndromes and the evolution of correlated behavior in zebrafish. Behavioral Ecology, 18, 556–562.

[ece33165-bib-0062] Muraco, J. J. , Aspbury, A. S. , & Gabor, C. R. (2014). Does male behavioural type correlate with species recognition and stress? Behavioral Ecology, 25, 200–205.

[ece33165-bib-0063] Nakagawa, S. , & Schielzeth, H. (2010). Repeatability for Gaussian and non‐Gaussian data: A practical guide for biologists. Biological Reviews, 85, 935–956. 20569253 10.1111/j.1469-185X.2010.00141.x

[ece33165-bib-0064] van Oers, K. , de Jong, G. , van Noordwijk, A. J. , Kempenaers, B. , & Drent, P. J. (2005). Contributions of genetics to the study of animal personalities: A review of case studies. Behaviour, 142, 1185–1206.

[ece33165-bib-0065] Petrosky, B. R. , & Magnuson, J. J. (1973). Behavioral responses of northern pike, yellow perch and bluegill to oxygen concentrations under simulated winterkill conditions. Copeia, 1, 124–133.

[ece33165-bib-0066] Pitcher, T. J. , & Parrish, J. (1993). The function of shoaling behaviour In Pitcher T. J. (Ed.), The behaviour of teleost fishes, 2nd ed. (pp. 363–439). London: Chapman & Hall.

[ece33165-bib-0067] Piyapong, C. , Krause, J. , Chapman, B. B. , Ramnarine, I. W. , Louca, V. , & Croft, D. P. (2010). Sex matters: A social context to boldness in guppies (*Poecilia reticulata*). Behavioral Ecology, 21, 3–8.

[ece33165-bib-0068] Plath, M. , & Schlupp, I. (2008). Parallel evolution leads to reduced shoaling behavior in two cave dwelling populations of Atlantic mollies (*Poecilia mexicana*, Poeciliidae, Teleostei). Environmental Biology of Fishes, 82, 289–297.

[ece33165-bib-0069] Plath, M. , Tobler, M. , & Riesch, R. (2015). Extremophile fishes: An introduction In Riesch R., Tobler M., & Plath M. (Eds.), Extremophile fishes. Ecology, evolution, and physiology of Teleosts in extreme environments (pp. 1–7). Heidelberg, New York: Springer.

[ece33165-bib-0070] Poeser, F. (2003). From the Amazon river to the Amazon molly and back again. Zoological Museum Amsterdam. IBED, Amsterdam University: Amsterdam, NL.

[ece33165-bib-0071] Polverino, G. , Ruberto, T. , Staaks, G. , & Mehner, T. (2016). Tank size alters mean behaviours and individual rank orders in personality traits of fish depending on their life stage. Animal Behaviour, 115, 127–135.

[ece33165-bib-0072] Pruitt, J. N. , Riechert, S. E. , & Jones, T. C. (2008). Behavioural syndromes and their fitness consequences in a socially polymorphic spider, *Anelosimus studiosus* . Animal Behaviour, 76, 871–879.

[ece33165-bib-0073] Réale, D. , Reader, S. M. , Sol, D. , McDougall, P. T. , & Dingemanse, N. J. (2007). Integrating animal temperament within ecology and evolution. Biological Reviews, 82, 291–318. 17437562 10.1111/j.1469-185X.2007.00010.x

[ece33165-bib-0074] Riesch, R. , Duwe, V. , Herrmann, N. , Padur, L. , Ramm, A. , Scharnweber, K. , … Plath, M. (2009). Variation along the shy‐bold continuum in extremophile fishes (*Poecilia mexicana, Poecilia sulphuraria*). Behavioral Ecology and Sociobiology, 63, 1515–1526.

[ece33165-bib-0075] Scharnweber, K. , Plath, M. , & Tobler, M. (2011b). Feeding efficiency and food competition in coexisting sexual and asexual livebearing fishes of the genus *Poecilia* . Environmental Biology of Fishes, 90, 197–205.

[ece33165-bib-0076] Scharnweber, K. , Plath, M. , Winemiller, K. O. , & Tobler, M. (2011a). Dietary niche overlap in sympatric asexual and sexual livebearing fishes (*Poecilia* spp.). Journal of Fish Biology, 79, 1760–1773. 22141886 10.1111/j.1095-8649.2011.03114.x

[ece33165-bib-0077] Schurmann, H. , & Steffensen, J. F. (1994). Spontaneous swimming activity of Atlantic cod *Gadus morhua* exposed to graded hypoxia at three temperatures. Journal of Experimental Biology, 197, 129–142. 9317484 10.1242/jeb.197.1.129

[ece33165-bib-0078] Seghers, B. H. (1973). Analysis of geographic variation in the antipredator adaptations of the guppy: Poecilia reticulata. unpublished, PhD Thesis, University of British Columbia.

[ece33165-bib-0079] Seghers, B. H. (1974). Schooling behavior in the guppy (*Poecilia reticulata*): An evolutionary response to predation. Evolution, 28, 486–489. 28564850 10.1111/j.1558-5646.1974.tb00774.x

[ece33165-bib-0080] Sih, A. , Bell, A. , & Johnson, J. C. (2004). Behavioral syndromes: An ecological and evolutionary overview. Trends in Ecology and Evolution, 19, 372–378. 16701288 10.1016/j.tree.2004.04.009

[ece33165-bib-0081] Timmermann, M. , Schlupp, I. , & Plath, M. (2004). Shoaling behaviour in a surface‐dwelling and a cave‐dwelling population of a barb *Garra barreimiae* (Cyprinidae, Teleostei). Acta Ethologica, 7, 59–64.

[ece33165-bib-0082] Utne‐Palm, A. C. (2002). Visual feeding of fish in a turbid environment: Physical and behavioural aspects. Marine and Freshwater Behaviour and Physiology, 35, 111–128.

[ece33165-bib-0083] Ward, A. J. W. , Thomas, P. , Hart, P. J. B. , & Krause, J. (2004). Correlates of boldness in three‐spined sticklebacks (*Gasterosteus aculeatus*). Behavioral Ecology and Sociobiology, 55, 561–568.

[ece33165-bib-0084] Whoriskey, F. G. , Gaudreault, A. , Martel, N. , Campeau, S. , & FitzGerald, G. J. (1985). The activity budget and behavior patterns of female threespine sticklebacks, *Gasterosteus aculeatus* . Naturaliste Canadien, 112, 113–118.

[ece33165-bib-0085] Wilson, A. D. M. , & Godin, J.‐G. J. (2009). Boldness and behavioral syndromes in the bluegill sunfish, *Lepomis macrochirus* . Behavioral Ecology, 20, 231–237.

[ece33165-bib-0086] Wright, D. , & Krause, J. (2006). Repeated measures of shoaling tendency in zebrafish (*Danio rerio*) and other small teleost fishes. Nature Protocols, 1, 1828–1831. 17487165 10.1038/nprot.2006.287

