# Supplementary material for: Predation risk and abiotic habitat parameters affect personality traits in extremophile populations of a neotropical fish (Poecilia vivipara)
Source: Ecol Evol. 2017 Jul 18;7(16):6570–81. doi: 10.1002/ece3.3165 (PMC5574810; doi:10.1002/ece3.3165)
Supplement: Supplementary file 1 [file ECE3-7-6570-s001.docx]

**Online supplementary material**

The following supporting information is available for the article online.

**
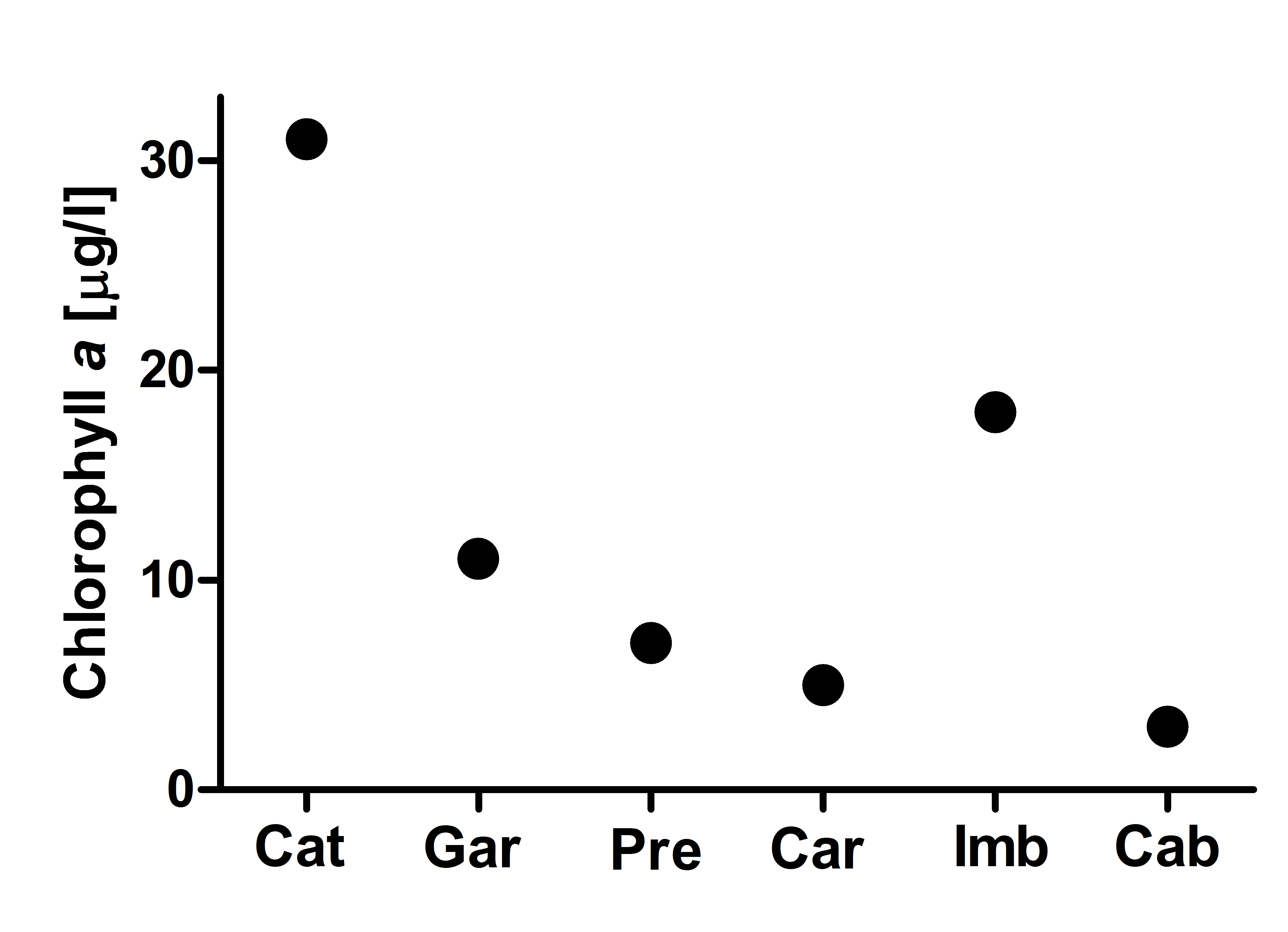
**

**Figure S1**

Clorophyll *a* concentrations vary among lagoons (Caliman *et al*. 2010). *Cat* = Catingosa, *Imb* = Imboassica, *Gar* = Garças, *Pre* = Preta, *Car* = Carapebus, *Cab* = Cabiúnas.

**Table S2**

Distribution of piscivorous fishes across the six lagoons investigated in this study (after Di Dario *et al*. 2013). “+” indicates presence of the respective fish species. *Imb* = Imboassica; *Cab* = Cabiúnas; *Car* = Carapebus; *Gar* = Garças; *Cat* = Catingosa; *Pre* = Preta.

| **Predatory species** | **Imb** | **Cab** | **Car** | **Gar** | **Cat** | **Pre** |
| --- | --- | --- | --- | --- | --- | --- |
| *Lycengraulis grossidens*  (Clupeiformes) | + | + | + | + |  |  |
| *Hoplerythrinus unitaeniatus*  (Clupeiformes) | + | + | + | + |  |  |
| *Hoplias malabaricus*  (Characiformes) | + | + | + | + |  | + |
| *Oligosarcus hepsetus*  (Characiformes) |  | + | + |  |  |  |
| *Synbranchus aff. marmoratus*  (Synbranchiformes) |  | + |  |  |  |  |
| *Centropomus parallelus*  (Perciformes) | + | + | + |  |  |  |
| *Centropomus undecimalis*  (Perciformes) | + | + | + |  |  |  |

**Table S3**

Results of generalized linear models combining both cohorts of focal fish (wild-caught and laboratory-maintained). Significant effects are indicated in bold typeface. Pronounced differences between both cohorts prompted separate analyses as reported in the main text.

| **Factor** | **Emergence time** | | **Activity** | | **Shoaling** | |
| --- | --- | --- | --- | --- | --- | --- |
|  | *χ*^2^ | *p* | *χ*^2^ | *p* | *χ*^2^ | *p* |
| Wild vs. laboratory | **65.10** | **< 0.001** | **17.12** | **< 0.001** | 2.36 | 0.12 |
| Predation | 0.002 | 0.97 | 1.98 | 0.16 | 1.45 | 0.23 |
| Turbidity | **5.57** | **0.020** | **19.90** | **< 0.001** | **38.02** | **< 0.001** |
| Salinity | 1.20 | 0.27 | 1.70 | 0.19 | **5.83** | **0.016** |
| DO | 1.67 | 0.20 | **4.22** | **0.040** | 0.49 | 0.49 |


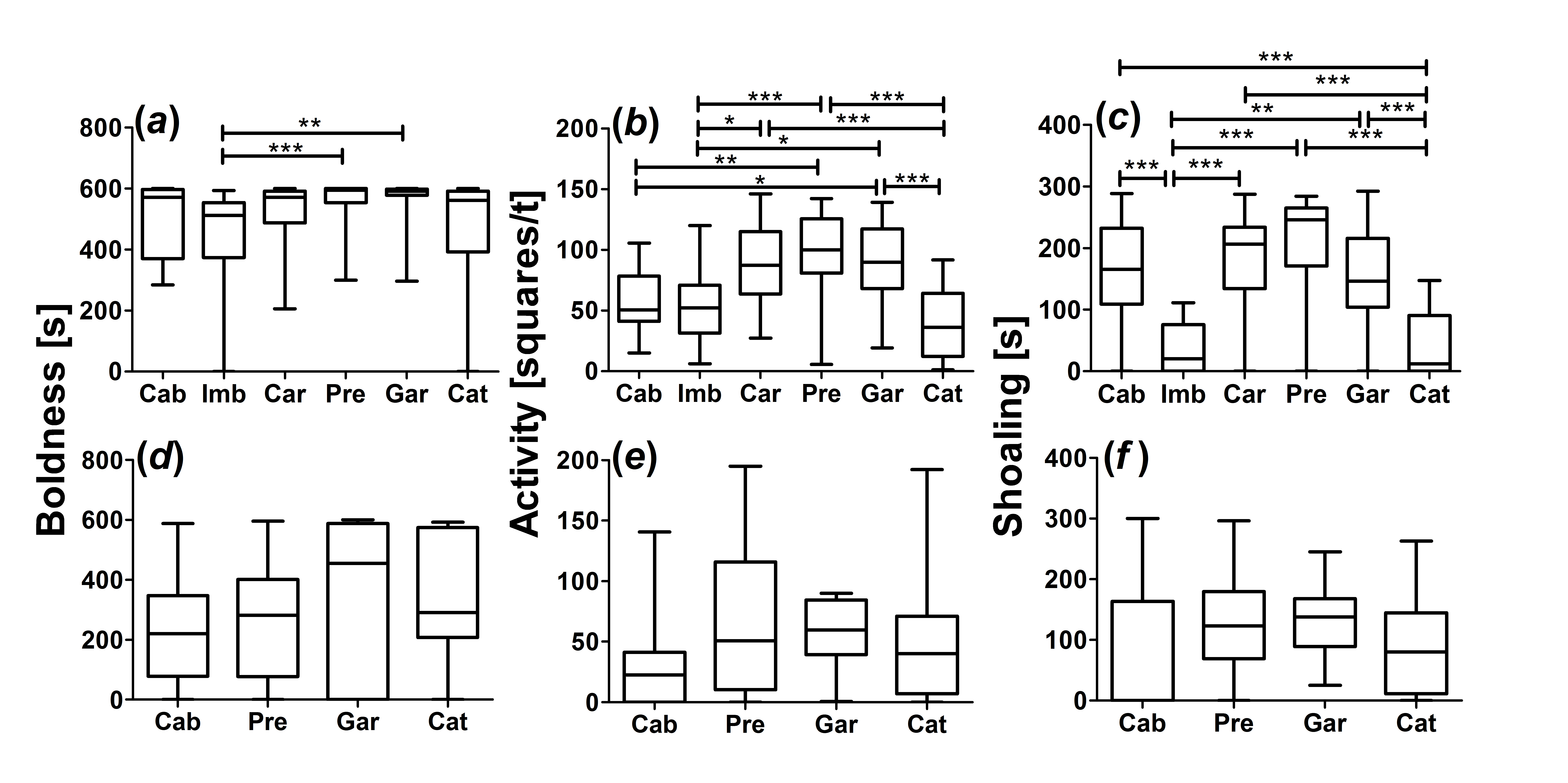


**Figure S4**

Boxplots showing medians (middle line), 25–75% ranges (boxes) and 5–95% ranges of the data from the three personality assessments (means from both measurements). (*a*) Boldness, (*b*) activity and (*c*) shoaling tendencies/sociability for wild-caught individuals and (*d*) boldness, (*e*) activity and (*f*) shoaling tendencies/sociability for laboratory-maintained fish. Significant differences refer to Dunn-Bonferroni all-pairwise *post hoc* tests (corrected for multiple comparisons), whereby * signifies *p* < 0.05; ** *p* < 0.01; and *** *p* < 0.001. *Cab*, Cabiunas; *Imb*, Imboassica; *Car*, Carapebus; *Pre*, Preta; *Gar*, Garças; *Cat*, Catingosa. Note that significant differences among fish from different lagoons disappeared after laboratory-maintainance.

**S5**

**Additional Discussion**

***Differences in behavioural consistency between wild-caught and laboratory-maintained fish***

Animal personality is, per definition, associated with a certain degree of consistency in behaviour that limits behavioural plasticity (or ‘flexibility’; Dall Houston & McNamara 2004; Réale *et al*. 2007; Wolf & Weissing 2010). This means that individual tendencies to act, for example, more or less aggressively, boldly, or in an explorative way, prevent individuals from expressing the full range of possible phenotypes along these behavioural axes (reviewed in Sih, Bell & Johnson 2004; Bell 2007). It has to be stressed though that the concept of animal personality and the assumption that individuals are able to show a certain degree of behavioural plasticity are not mutually exclusive as long as differences between individuals are maintained over time or/and across contexts (Dingemanse *et al*. 2010). If behavioural plasticity is associated with costs (Dall *et al*. 2004), a trade-off is predicted to arise between plastic behaviour (which allows better adjustment to changing environmental conditions) and consistent behaviour (which reduces costs associated with behavioural plasticity). Following this idea, selection should lead to a reduction/loss of behavioural plasticity in populations living under rather stable environmental conditions.

In our present study, we found fish from the laboratory-maintained cohort (benign and stable environmental conditions) to show a higher repeatability in behaviour than wild-caught individuals of the same lagoons. Stronger behavioural consistency in the laboratory-maintained can only be interpreted as a signal of non-heritable behavioural adjustment (since we tested only fish of the same generation) and plasticity could be reduced by the absence of novel/changing stimuli like they are experienced under natural conditions.

**References**

Bell, A.M. (2007) Future directions in behavioural syndromes research. Proceedings of the Royal Society B, 274, 755-761.

Caliman, A., Carneiro, L.S., Santangelo, J.M., Guariento, R.D., Pires, A.P.F., Suhett, A.L., Quesado, L.B., Scofield, V., Fonte, E.S., Lopes, P.M., Sanches, L.F., Azevedo, F.D., Marinho, C.C., Bozelli, R.L., Esteves, F.A. & Farjalla, V.F. (2010) Temporal coherence among tropical coastal lagoons: a search for patterns and mechanisms. Brazilian Journal of Biology, 70, 803-814.

Dall, S.R.X., Houston, A.I. & McNamara, J.M. (2004) The behavioural ecology of personality: consistent individual differences from an adaptive perspective. Ecology Letters, 7, 734-739.

Di Dario, F., Petry, A.C., de Souza Pereira, M.M., Mincarone, M.M., Soares Agostinho, L., Martins Camara, E., Pellegrini Caramaschi, E. & de Britto, M.R. (2013) An update on the fish composition (Teleostei) of the coastal lagoons of the Restinga de Jurubatiba National Park and the Imboassica Lagoon, northern Rio de Janeiro State. Acta Limnologica Brasiliensia, 25, 257-278.

Dingemanse, N.J., Kazem, A.J.N., Réale, D. & Wright, J. (2010) Behavioural reaction norms: animal personality meets individual plasticity. Trends in Ecology and Evolution, 25, 81-89.

Réale, D., Reader, S.M., Sol, D., McDougall, P.T. & Dingemanse, N.J. (2007). Integrating animal temperament within ecology and evolution. Biological Reviews, 82, 291-318.

Sih, A., Bell, A. & Johnson, J.C. (2004) Behavioral syndromes: an ecological and evolutionary overview. Trends in Ecology and Evolution, 19, 372-378.

Wolf, M., Weissing, F.J. (2010) An explanatory framework for adaptive personality differences. Philosophical Transactions of the Royal Society B, 365, 3959-3968.
